# The burden of diarrhea, etiologies, and risk factors in India from 1990 to 2019: evidence from the global burden of disease study

**DOI:** 10.1186/s12889-022-12515-3

**Published:** 2022-01-13

**Authors:** Deepak Kumar Behera, Sanghamitra Mishra

**Affiliations:** 1 Faculty of Health Economics, Department of Commerce, Manipal Academy of Higher Education (MAHE), Manipal, Karnataka 576 104 India; 2 The Medics, Angul, Odisha 759122 India

**Keywords:** Burden of diarrhea, Etiology of diarrhea, Risk factor for diarrhea, Burden of disease, India

## Abstract

**Background:**

This study aims to measure the burden of diarrhea in India and analyze the trend of mortality associated with it for the past 30 years. We also intend to find the prevailing etiology and risk factors associated with diarrheal mortality in India.

**Methods:**

The study has used the latest round of Global Burden of Disease (GBD) study-2019. GBD data is available across age groups and gender-wise over the period from 1990 to 2019. The study has identified 13 etiologies for the cause of diarrhea deaths and 20 risk factors to analyze the burden of disease.

**Results:**

Our study shows, childhood diarrhea has declined over the years significantly, yet contributes to a larger share of DALYs associated with the disease. Among all the death cases of Diarrhea, in 2019, the most prevalent disease-causing pathogen is found to be Campylobacter. But Adenovirus is the major contributor to childhood diarrheal deaths. Though the burden of diarrhea is declining over the period, still there is a need to progress the interventions to prevent and control diarrhea rapidly to avoid the huge number of deaths and disabilities experienced in India.

**Conclusions:**

Consumption of safe and clean water, proper sanitation facility in every household, required nutrition intake by mother and child, safe breastfeeding and stool disposal practices and careful case management, rotavirus vaccination are some of the effective interventions to be implemented all over the country. Further, evidence-based policies should be made and implemented to sustain diarrhea prevention programs.

**Supplementary Information:**

The online version contains supplementary material available at 10.1186/s12889-022-12515-3.

## Background

The epidemiological transition from communicable diseases to non-communicable diseases has shifted the attention of public health researchers accordingly in recent years. Yet, the morbidity, mortality, and disability associated with communicable diseases, or so-called primitive diseases, are still significant. Among all, Diarrhea is one of the top 10 diseases to contribute to global DALY, even in recent years. It is found to be one of the most common diseases among children under-5 years [1]. Diarrhea is a disease that has different kinds to it, according to ICD-10, such as infective neonatal diarrhea, unspecified diarrhea, functional diarrhea, non-infective neonatal diarrhea, and irritable bowel syndrome with diarrhea, etc. [2]. Along with the diseased person, it also affects the well-being of the ICU health workers due to the increasing workload. Heidegger et al. say diarrhea episodes have a huge impact on human-related costs and so on the financial burden in ICU [3].

In low and middle-income countries, diarrhea possess a great threat to the population and its economy. In these countries, the prevalence of diarrheal disease seems to be rather increasing with time while poverty is a major factor influencing this trend. Ahs et al. argue that current diarrhea-related mortality among children is a result of inequity in resource distribution such as insufficient health care staff, unequal access to healthcare services [4]. Not having enough knowledge on care-seeking behavior and case management practices lead to poor case management of childhood diarrhea in LMICs, hence need improvement [5]. Reportedly, harmful practices such as restrictions of fluid or breast milk during diarrhea, administration of modern medicine in the wrong manner in the process of diarrhea management rather have negative health outcomes and possibly create conflicts with WHO treatment regimen [6]. Adapting healthy practices such as building sanitation infrastructure, consumption of safely managed water, and reducing diarrhea-associated mortality in high-income countries is evident [7].

Mortality due to diarrheal disease has been declining due to various preventive measures taken at an individual and national level, yet the incidence remains constant. Apart from this, cured individuals also contribute to various other kinds of public health burdens such as impaired cognitive development, reduced immune response, growth faltering, and death, at times [8, 9]. Along with LMICs, even HICs have to carry huge public health and economic burden associated with it. One of the most developed countries, the United States of America, lost approximately 6 billion US dollars/ year due to medical expenses and resulting unproductivity [10].

Even after being one of the leading causes of mortality and morbidity in the world, data associated with diarrheal diseases are limited [1]. As for the etiological causes, although there are many e.g., viruses, bacteria, parasites, etc., there is no gold standard for it [11]. Risk factors that can make a population more vulnerable to diarrhea can be environmental or behavioral, yet differ among individuals, populations, countries, and geographies.

This study aims to measure the burden of diarrhea in India in 2019 and analyze the trend of mortality associated with it for the past 30 years. We also intend to find the prevailing etiology and risk factors associated with diarrheal deaths in India in the year 2019. The rest of the paper is divided into four sections. Section 2 discusses methods. Section 3 contains the results. Section 4 critically discusses the results. Finally, section 5 concludes the study.

## Methods

The study has used the latest round of GBD study 2019, which is a comprehensive database related to communicable diseases, non-communicable diseases, and injuries over the period from 1990 to 2019 across the 204 country samples [12]. The latest round of GBD survey-2019 provides information about 369 diseases and injuries related incidence, death, and disability adjust life years (DALYs). Additionally, it provides information about 87 risk factors of the cause of disease and etiologies. This data is available across age groups and gender-wise over the period from 1990 to 2019. In this study, we have analyzed the data related to Diarrhea disease – number of deaths, mortality rate, DALYs rate, associated risk factors, etiologies in India. The study has identified 13 etiologies for the cause of diarrhea deaths (see Table 2) and 20 risk factors (see Table 3) to analyze the burden of disease. The analysis has been done across the period, across age groups, and across gender in India. Additional file 1: Table A1 shows the indicators and measurement criteria have used in the study.

## Results

### Burden of diarrhea – number of deaths, mortality, and DALYs

Table 1 presents the number of diarrhea deaths and mortality rate (per 100,000 population) in 2019 in India for each age group and sex. The latest data in 2019 shows, the total number of deaths of all ages is 632,344 in 2019 (95% uncertainty interval; 358,561–1,056,036), and the mortality rate is 45 per 100,000 population (95% uncertainty interval; 25–75). Most deaths occurred in the older age group – 70+ years followed by 50–69 years in 2019. It has estimated that the mortality rate 682 (95% uncertainty interval: 362–1144) for 70+ years and 62 95% (uncertainty interval: 31–118) for 50–69 years in 2019. Similarly, the number of child deaths in the age group - Under 5 is 55,309 in 2019 (95% uncertainty interval: 39882–73,621), and the mortality rate is 47 per 100,000 population (95% uncertainty interval; 34–62).

**Table 1:** The number of diarrhea deaths and mortality rate (per 100,000 population) in 2019 in India for each age group and sex

Age	Sex	Number of Deaths	Mortality Rate
All Ages	Male	230,468.8 (142738–482,412.4)	32.31 (20.01–67.64)
All Ages	Female	401,876 (166,196.4–793,477.5)	59.31 (24.53–117.11)
All Ages	Both	632,344.7 (358561–1,056,036)	45.46 (25.78–75.93)
Under 5	Male	22,392.69 (16,201.54–29,976.6)	36.53 (26.43–48.90)
Under 5	Female	32,916.93 (22,665.14–46,353.51)	59.01 (40.63–83.10)
Under 5	Both	55,309.61 (39,882.77–73,621.29)	47.24 (34.06–62.88)
5–14 years	Male	6742.824 (3555.182–14,735.31)	4.97 (2.62–10.87)
5–14 years	Female	9609.717 (3429.244–19,759.56)	7.77 (2.77–15.98)
5–14 years	Both	16,352.54 (8494.022–27,826.46)	6.31 (3.27–10.74)
15–49 years	Male	22,323.31 (11,883.54–56,059.29)	5.70 (3.03–14.33)
15–49 years	Female	28,228.08 (9025.635–62,731.19)	7.66 (2.45–17.02)
15–49 years	Both	50,551.39 (25,651.88–94,949.45)	6.65 (3.37–12.50)
50–69 years	Male	47,983.77 (25,751.48–117,218)	48.59 (26.08–118.71)
50–69 years	Female	76,579.09 (24,773.43–24,773.43)	76.79 (24.84–171.11)
50–69 years	Both	124,562.9 (62,219.35–234,874.3)	62.76 (31.35–118.35)
70+ years	Male	131,026.2 (78,186.29–271,554.5)	495.20 (295.49–1026.31)
70+ years	Female	254,542.2 (98,380.17–506,093.9)	846.84 (327.30–1683.73)
70+ years	Both	385,568.3 (204,662.6–646,591.9)	682.21 (362.12–1144.06)

Note: Parenthesis denotes 95% uncertainty interval of lower and upper limits

Table 1 also presents gender-wise differences in diarrhea-related deaths and mortality in India in 2019. It has been shown that the number of deaths of males and females is 32.31 (95% uncertainty interval: 20.01–67.64) and 59.31 (95% uncertainty interval: 24.53–117.11) respectively of all age groups. These mortality differences between males and females are also exhibited in other age categories as well and it is clearly shown that females are more prone to diarrhea-related deaths than males. The differences in mortality are more widened in the age group 70+ years where the female mortality rate is 846.84 (95% uncertainty interval: 327.30–1683.73) and 495.20 (95% uncertainty interval: 295.49–1026.31). Similarly, child mortality is more prone due to the cause of diarrhea, and the gender difference between a male and female child is visible in Table 1.

**Fig. 1:**
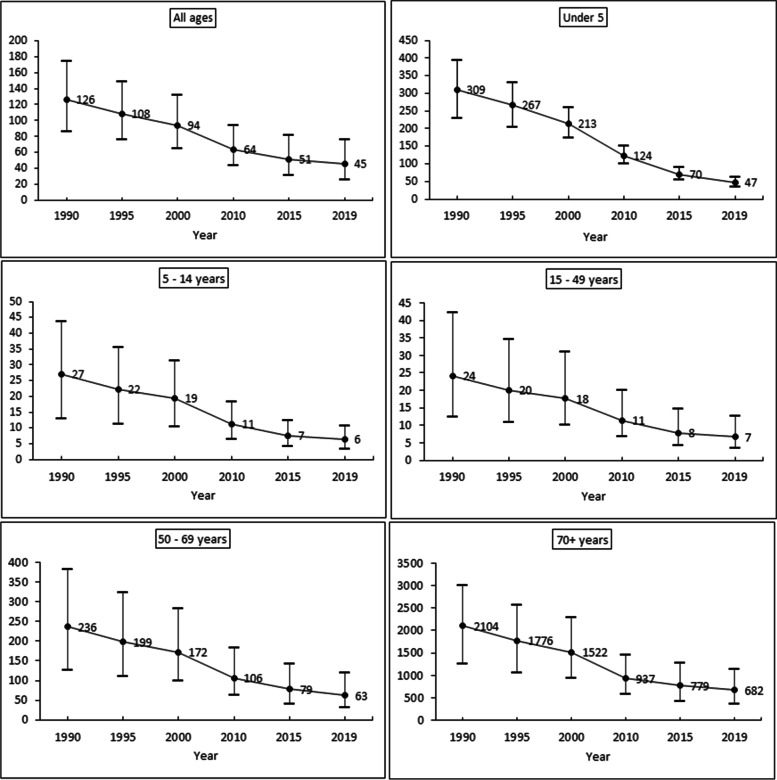
Diarrhea mortality rate (per 100,000 population) by age, 1990–2019 in India. Note: Point represent mean estimates and error bars represents 95% uncertainty interval of the lower and upper limit

Figure 1 shows the time-series trends in Diarrhea mortality rate (per 100,000 population) by age in India from 1990 to 2019. All ages: The mortality rate has been declined from 126 in 1990 to 45 in 2019. Under − 5: The mortality rate has been declined from 309 in 1990 to 47 in 2019. Five to fourteen years: The mortality rate has been declined from 27 in 1990 to 6 in 2019. Fifteen to forty-nine years: The mortality rate has been declined from 27 in 1990 to 6 in 2019. Fifty to sixty-nine years: The mortality rate has been declined from 236 in 1990 to 63 in 2019. 70+ years: The mortality rate has been declined from 2104 in 1990 to 682 in 2019. The overall analysis of Fig. 1 depicts that the deaths of diarrhea have been declined over the period across all age groups in India but still it plays a major communicable disease in India.

**Fig. 2:**
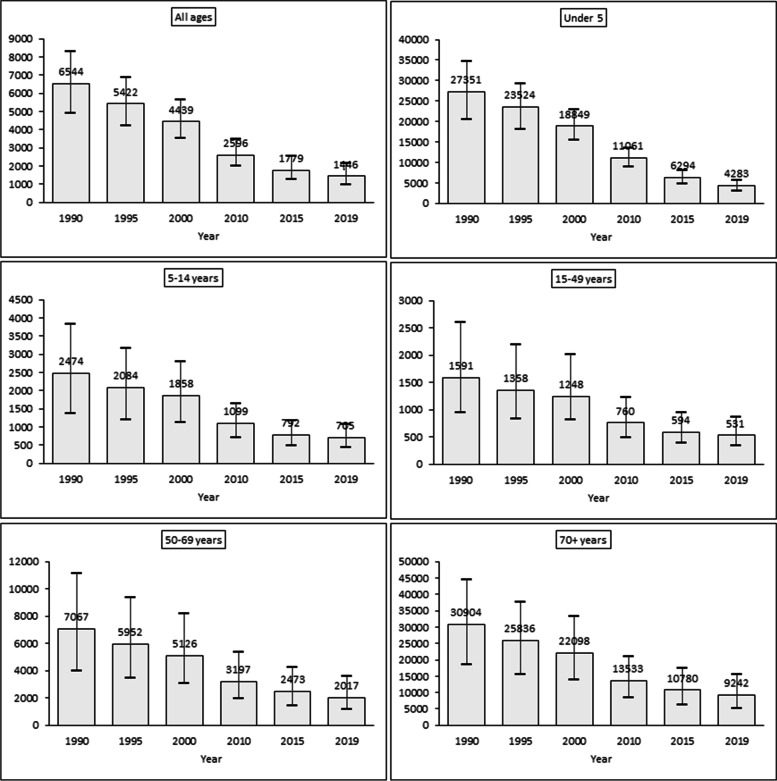
Diarrhea DALYs rate (per 100,000 population) by age, 1990–2019 in India. Note: Point represent mean estimates and error bars represents 95% uncertainty interval of the lower and upper limit

Figure 2 shows the time-series trends in Diarrhea DALYs rate (per 100,000 population) by age in India from 1990 to 2019. All ages: The DALYs rate has been declined from 6544 in 1990 to 1446 in 2019. Under − 5: The DALYs rate has been declined from 27,351 in 1990 to 4283 in 2019. Five to fourteen years: The DALYs rate has been declined from 2474 in 1990 to 705 in 2019. Fifteen to forty-nine years: The DALYs rate has been declined from 1591 in 1990 to 531 in 2019. Fifty to sixty-nine years: The DALYs rate has been declined from 7067 in 1990 to 2017 in 2019. 70+ years: The DALYs rate has been declined from 30,904 in 1990 to 9242 in 2019. The overall analysis of Fig. 2 depicts that the DALYs rate of diarrhea has been declined over the period across all age groups in India but still around 705 persons of all ages are living with disabilities in 2019. Similarly, the under-5 and older age (i.e., 70+ years) are more DALYs rate that is 4283 and 9242 persons in 2019 respectively.

### Etiologies for the cause of diarrhea disease and deaths

Table 2 represents etiologies for the cause of diarrhea disease and the number of deaths among under-5 and all ages in India, 2019. We have found that there is 13 etiology that causes diarrhea in India. We have found that Adenovirus is the most prominent pathogen for the cause of diarrhea among children, followed by Campylobacter and Cryptosporidium. Our result shows that around 8864 deaths occurred due to Adenovirus whereas around 8435 for Campylobacter and 5074 for Cryptosporidium in 2019. Similarly, the most pertinent pathogen for the cause of diarrhea for all ages is Campylobacter followed by Cryptosporidium and Norovirus. Our results show that around 44,353 deaths occurred due to Campylobacter whereas around 36,007 for Cryptosporidium and 29,924 for Norovirus in 2019.

**Table 2:** Etiologies for the cause of diarrhea disease and number of deaths among under-5 and all ages in India, 2019

Etiology	Number of Deaths	
	Under 5	All Ages
Cholera	308.20 (168.86–529.62)	3832.13 (2241.65–6253.05)
Non-typhoidal Salmonella	2061.21 (315.36–5309.76)	9143.28 (315.36–35,931.43)
Shigella	4962.37 (1679.89–11,247.34)	24,474.21 (8457.54–53,447.86)
Enteropathogenic E coli	1556.12 (684.65–2950.12)	3393.35 (1595.33–6108.74)
Enterotoxigenic E coli	2669.03 (1059.49–5673.76)	22,668.51 (9418.76–48,347.06)
Campylobacter	8435.41 (2991.94–17,552.2)	44,353.98 (10,550.04–116,332.8)
Entamoeba	3347.33 (903.44–8623.98)	11,199.08 (3331.32–28,713.4)
Cryptosporidium	5074.18 (836.74–15,270.26)	36,007.17 (4993.42–125,954.9)
Rotavirus	2059.74 (767.51–4378.68)	4378.68 (17,500.49–91,197.18)
Aeromonas	793.62 (285.88–1673.29)	3025.27 (1340.69–5877.25)
*Clostridium difficile*	129.76 (62.78–243.66)	1150.95 (724.41–1690.44)
Norovirus	2317.06 (574.28–5613.38)	29,924.36 (3711.67–80,822.81)
Adenovirus	8864.36 (4317.97–16,036.32)	20,916.49 (11,657.05–33,668.17)

Note: Point represent mean estimates and error bars represents 95% uncertainty interval of the lower and upper limit

### Risk factors for the cause of diarrhea disease and mortality

Table 3 shows the risk factors for the cause of diarrhea disease and mortality rate (per 100,000) among under-5 and all ages in India, 2019. We have found many environmental and behavioral risk factors that lead to diarrhea. The environmental factors include unsafe water, sanitation, and handwashing; and air pollution (i.e., ambient particulate matter pollution and household air pollution from solid fuels). The behavioral factors include suboptimal breastfeeding; malnutrition; vitamin A deficiency. Our result shows that major risk factors of diarrhea-related child mortality are unsafe water sources followed by child wasting and unsafe sanitation. It is shown that the under-5 diarrhea-specific mortality rate is 43 (95% uncertainty interval: 30–59) due to unsafe water, sanitation, and handwashing factors. Similarly, the mortality rate is 44 (95% uncertainty interval: 32–59) due to child and maternal malnutrition factors. Our result shows that major risk factors of diarrhea-specific mortality of all ages are unsafe water sources. It is shown that the all-ages diarrhea-specific mortality rate is 41 (95% uncertainty interval: 23–69) due to unsafe water, sanitation, and handwashing factors. Overall analysis shows that unsafe water, sanitation, and handwashing practices are the major risk factors for diarrhea-specific mortality in India of all ages as well as under-5 in India. But risk factors such as Suboptimal breastfeeding, Child growth failure, and low birth weight, and Vitamin A deficiency are the pertinent factors for the cause of diarrhea-specific mortality for children (i.e., Under 5) in India.

**Table 3:** Risk factors for the cause of diarrhea disease and mortality rate (per 100,000) among under-5 and all ages in India, 2019

S.N.	Risk factor	Mortality rate (Under 5)	Mortality rate (All ages)
A	Environmental risks	43.50 (30.80–59.30)	41.31 (23.51–69.19)
1	Unsafe water, sanitation, and handwashing	43.36 (30.71–59.14)	41.30 (23.50–69.18)
1.1	Unsafe water source	38.60 (26.01–53.43)	36.55 (19.49–60.92)
1.2	Unsafe sanitation	22.36 (15.81–29.96)	20.93 (11.86–35.28)
1.3	No access to a handwashing facility	9.82 (6.72–13.76)	8.82 (4.77–14.96)
2	Air pollution	1.80 (1.17–2.68)	0.15 (0.10–0.23)
2.1	Ambient particulate matter pollution	0.95 (0.55–1.53)	0.08 (0.05–0.13)
2.2	Household air pollution from solid fuels	0.85 (0.50–1.37)	0.07 (0.04–0.11)
B	Behavioral risks	44.36 (32.08–59.17)	3.73 (2.70–4.98)
1	Child and maternal malnutrition	44.36 (32.08–59.17)	3.73 (2.70–4.98)
1.1	Suboptimal breastfeeding	8.78 (5.87–12.94)	0.74 (0.49–1.09)
1.1.1	Non-exclusive breastfeeding	8.30 (5.44–12.42)	0.70 (0.46–1.05)
1.1.2	Discontinued breastfeeding	0.58 (0.18–1.09)	0.05 (0.09–0.02)
1.2	Child growth failure	36.64 (26.69–48.57)	3.08 (2.25–4.09)
1.2.1	Child underweight	6.46 (4.38–9.32)	0.54 (0.37–0.78)
1.2.2	Child wasting	35.46 (25.49–47.57)	2.99 (2.15–4.00)
1.2.3	Child stunting	6.10 (2.03–11.72)	0.51 (0.17–0.99)
1.3	Low birth weight and short gestation	6.91 (4.60–10.43)	0.58 (0.39–0.88)
1.3.1	Short gestation for birth weight	4.10 (2.72–6.13)	0.34 (0.23–0.52)
1.3.2	Low birth weight for gestation	6.75 (4.48–10.16)	0.57 (0.38–0.86)
1.4	Vitamin A deficiency	1.02 (0.17–2.15)	0.09 (0.01–0.18)
1.4.1	Zinc deficiency	0.30 (0.06–0.68)	0.02 (0.01–0.06)

Note: Point represent mean estimates and error bars represents 95% uncertainty interval of the lower and upper limit

## Discussion

### Changing pattern of diarrhea cases in India

As our study suggests the overall morbidity and mortality associated with diarrheal disease has declined from 1990 till 2019, a similar pattern of diarrhea cases has been observed in India and different parts of the world by several public health researchers so far.

Troeger et al. have studied diarrhea burden in different regions of the world and found that although the disease burden associated with diarrhea has been declining over the years, it still is one of the biggest threats among children under 5 years and the elderly population specifically i.e., more than 70 years old [13]. In 2015, although children under 5 years had the highest number of deaths due to diarrhea among all age groups in Eastern Mediterranean Region, 17 out of 22 countries showed higher mortality among the elderly (70+) population than any other age group [14]. Similarly, this study also depicts the highest mortality among the elderly population, making them more vulnerable.

As the result of our study shows, childhood diarrhea has declined over the years significantly, yet contributes to a larger share of DALYs associated with the disease. In India, as most of the low-and middle- income countries, diarrheal proportional mortality has always been high among children even after a steady decline in childhood diarrhea deaths [15]. Being the 2nd largest cause of the under-5 mortality rate of Ethiopia, in 2013 since 1990, it shows diarrhea not only causes disability and discomfort in the younger population but also leads to deaths for a very long period [16].

### Prevailing pathogens and risk factors of diarrhea

Among all the death cases of Diarrhea, in 2019, the most prevalent disease-causing pathogen is found to be Campylobacter. But, Adenovirus is the major contributor to childhood diarrheal deaths. While most of the existing literature from all over the world suggests Rotavirus being the major cause of childhood diarrhea, the number of deaths associated with it among the children under 5 years in our study is relatively much lesser i.e., 2059.74 per 100,000 population, compared to Adenovirus i.e., 8864.36 per 100,000 populations. Even though Rotavirus does not cause the greatest number of deaths, it still is one of the major fatal pathogens. Reportedly, these deaths can be reduced by promoting and implementing the rotavirus vaccine in the country. By averting more than 28,000 deaths of young children around the globe, in 2016, the rotavirus vaccine has proved its efficiency [17].

As our study indicates that unsafe water, sanitation, and handwashing practices with child growth failure, LBW has a major role in increased morbidity and mortality associated with childhood diarrhea (or diarrhea in general), similarly, Lakshminarayanan and Jayalakshmy have also found a significant association of such risk factors with childhood diarrhea along with other types of risk factors. These include – age, socio-economic status, unhealthy breastfeeding practices, low birth weight, having younger siblings, etc. [15]. This finding is also parallel with many other existing public health literature by various researchers. In rural Mozambique, washing hands facilities with child stool disposal system has a noticeable impact on reduced risk of middle to severe diarrhea among children, and not providing fresh drinking water has contributed to a higher risk of childhood diarrhea [18]. In a study regarding diarrhea in urban slums of India, it was also seen that the incidence of diarrhea is relatively higher among children of poor (lower SES) strata [19]. While most of the discussion about the risk factor of diarrhea is concentrated around the consumption of unsafe water and poor sanitation, predisposing factors such as unsafe medium of drinking water conveyance, poor condition of water tanks at houses, water handling practices at households, improper disposal of wastewater and fecal matter, etc. are essential to look for to prevent diarrhea [20, 21]. Surprisingly, although not in our study, many studies have shown that the attack rate of diarrhea is higher among families with more than one child under 5 years of age than families with a single child [22].

Apart from these aforementioned factors, the knowledge, attitude, and behavior of mothers have also a significant impact on diarrhea among the younger population. Singh et al. have found a positive correlation between the educational status of a mother with the incidence of diarrheal disease [23]. Further, the literacy status of the mother can also be associated with the risk behaviors they practice at their households such as not washing hands before feeding the child, washing hands without soap, improper disposal of stools, etc. [24]. In our study, as well, the mortality rate associated with diarrhea among the under-5 cohort differs due to different types of breastfeeding behavior shown by mothers. As the children are not capable of taking the decision, hence, parents are needed to make healthy decisions for their children and the family, as a whole.

Child and maternal malnutrition are a well-acknowledged risk factor of diarrhea for ages and a similar result is also observed in our study. But in some studies, the frequency of diarrhea was found to be not significantly related to the nutritional status of the children [25]. Similarly, Ganguly et al. found that although malnutrition is significantly associated with diarrhea, there is no significant association with lack of breastfeeding, low literacy status of the mother, and consumption of untreated drinking water [26]. These findings suggest that although there are tons of literature in place, still there is a need for more research regarding risk factors of diarrhea as it may differ between countries, regions, populations, or age groups.

## Conclusions

Using global burden of disease data, this study was examined the burden of diarrhea, etiologies, and risk factors in India over the last 30 years from 1990 to 2019. We have found that most deaths occurred in the older age group – 70+ years followed by 50–69 years in 2019. Study results also show that childhood diarrhea has declined over the years significantly. Among all the death cases of Diarrhea, in 2019, the most prevalent disease-causing pathogen is found to be Campylobacter. But Adenovirus is the major contributor to childhood diarrheal deaths. Though the burden of diarrhea is declining over the period, still there is a need to progress the interventions to prevent and control diarrhea rapidly to avoid the huge number of deaths and disabilities experienced in India. Consumption of safe and clean water, proper sanitation facility in every household, required nutrition intake by mother and child, safe breastfeeding and stool disposal practices and careful case management, rotavirus vaccination are some of the effective interventions to be implemented all over the country. Along with these, more public health research regarding prevalence, etiology, and risk factors should be carried out in the future. Further, evidence-based policies should be made and implemented to sustain diarrhea prevention programs.

## Supplementary Information

**Additional file 1: Table A1.** Definition of Variables and measurement.

## Data Availability

Data is available in the public domain for research purposes and not for commercial use. Data can be obtained from the open access repository of the Institute of Health Metrics Evaluation (IHME).

## Abbreviations

DALYs: Disability Adjust Life Years
GBD: Global Burden of Disease
HICS: Hospital Incident Command System
IHME: Institute for Health Metrics and Evaluation
ICD: International Classification of Diseases
ICU: Intensive Care Unit
LMICs: Low-and Middle Income Countries
WHO: World Health Organization
