# Supplementary material for: The burden of diarrhea, etiologies, and risk factors in India from 1990 to 2019: evidence from the global burden of disease study
Source: BMC Public Health. 2022 Jan 13;22:92. doi: 10.1186/s12889-022-12515-3 (PMC8759196; doi:10.1186/s12889-022-12515-3)
Supplement: Supplementary file 1 — Additional file 1: Table A1. Definition of Variables and measurement. [file 12889_2022_12515_MOESM1_ESM.docx]

Additional file 1

Table A1. Definition of Variables and measurement

| Measure | Number | Percent | Rate |
| --- | --- | --- | --- |
| Deaths | Number of deaths in the population | The proportion of deaths for a particular cause relative to deaths from all causes | Deaths per 100,000 population |
| Disability-adjusted life years (DALYs) | Number of DALYs in the population | The proportion of DALYs for a particular cause relative to DALYs for all causes | DALYs per 100,000 population |
| Incidence | Number of new cases in the population | The proportion of new cases of a particular cause relative to cases from all causes | New cases per 100,000 population |

Source: Author’s estimation from GBD -2019 (IHME, 2020)
